# The Teeth of the Ancient Greeks

**Published:** 1880-11

**Authors:** 


### THE TEETH OF THE ANCIENT GREEKS.

One of the most remarkable features of the discovery of the band of Thebans who fell at Chæronea is that, according to the report, all the teeth of each member of the sacred band are sound and complete. Either these gallant patriots were exceptionally lucky, or the condition of teeth in old Greece was enviably different from that of later and more degenerate days. The Romans were well acquainted with the evils that attend on the possession of teeth, and had some considerable knowledge of the use of gold in counteracting these evils If we remember rightly, an exception to the rule of not burying precious objects with departed Romans was made in favor of the gold that had been used for stopping teeth. We moderns may compare favorably with the Romans in the skill of our dentists, but we cannot pretend to rival the defenders of Thebes in their superiority to the necessity for these gentlemen. Rare indeed are the happy mortals of to day who can truly boast that their teeth are in the perfect condition that nature intended, and that the craft of the dentist has never been employed upon them. It would be a difficult task to select from our army, or any modern army, 300 men with teeth as sound as those of the Theban warriors are reported to be.—*London News.*

It does not require a very great stretch of credulity to accept in the main, the above statement. Many of the ancient nations, selected in early life, the best physical specimens of their race for warriors. These were cared for, and trained through life, in such a manner as to secure the most perfect physical developement Every thing was done to promote this end; all adverse conditions and circumstances were avoided. Hereditary peculiarities, defects and tendencies were, doubtless, closely scrutinized and investigated. In ancient times war was more a matter of physical force and power than at the present; hence, the importance of such careful selection and culture.

But, aside from the arts of war, great attention was given among the Greeks to physical culture.

Curtis, in his history of Greece, says, "Popular culture, more than anything else distinguishes the Greeks from the barbarians of modern times — the idea of comprehending body and soul in an equal measure. The Greeks could not conceive of a healthy mind in a sick body, of a serene soul in a neglected and unworthy covering. The just balance of a spiritual and physical power the harmonious development of all natural powers and impulses, formed the task of education for the Hellenes; and hence a vigorous agility and elasticity of limb, endurance in running and in the contest, a firm and light step a free and sure bearing, freshness of health, a clear and animated eye. The music and gymnastic arts were inseparably connected, in order to train from generation to generation the youth, and give a healthy body and soul. This double education was not left to the arbitrary decision of the single familes, but was ordered and fostered by the state. It was impossible to imagine a Hellenic city without public gymnasia, abounding in large and sunny spaces for exercise. Whoever wished to claim authority and influence among his fellow citizens, must have spent the greater part of his time, up to the attainment of the maturity of manhood in the gymnasia."

Thus it was that wise regulations for physical development were made and enforced, and the result was fine bodily development. When this is taken into account, together with the fact that great care was exercised in the selection of soldiers, it is no matter of surprise that so many sound and complete sets of teeth were found, as stated in the above quotation from the London News.

